# Frantz Tumor: A Case Report of Solid Pseudopapillary Tumor of Pancreas

**DOI:** 10.7759/cureus.41698

**Published:** 2023-07-11

**Authors:** Nadezhda Stefanova, Turgay Kalinov, Nikola Kolev, Emilian Kalchev

**Affiliations:** 1 Department of General and Clinical Pathology, Forensic Medicine and Deontology, Medical University of Varna, "St. Marina" University Hospital, Varna, BGR; 2 Department of General Surgery, Medical University of Varna, "St. Marina" University Hospital, Varna, BGR; 3 Department of Diagnostic Imaging and Radiotherapy, Medical University of Varna, "St. Marina" University Hospital, Varna, BGR

**Keywords:** distal pancreatectomy (dp), pet scans, pancreas lesion, pancreatic cancer, franz tumor, case report, pseudopapillary tumor

## Abstract

The solid pseudopapillary tumor (SPT) is a rare pancreatic lesion that usually affects young and middle-aged patients and has a female predominance and low malignant potential. The exact histogenesis of this tumor is still unclear.

We present the case of a 60-year-old female patient with occasional abdominal pain. Positron emission tomography/computed tomography (PET/CT) and magnetic resonance imaging (MRI) revealed a tumor mass in the pancreatic tail. Distal pancreatectomy and splenectomy were performed. The result from the pathology report was solid pseudopapillary neoplasm (SPN). The patient underwent four cycles of adjuvant chemotherapy with gemcitabine, which she tolerated well without complaints. A control computed tomography (CT) scan and PET/CT of the abdomen (five months after the operation) showed a cystic lesion suspicious for local recurrence in the pancreatic tail during the follow-up period. The patient underwent a second surgery operation. Subsequent histological examination showed chronic indurative pancreatitis, areas with steatonecrosis, lipogranulomas, and fibrosis without evidence of relapse.

SPT is a rare pancreatic tumor that most commonly affects young women. Although the tumor has locally aggressive characteristics, the prognosis is excellent after surgical excision. Our case emphasizes that this tumor can occur not only in young women but also in older patients. Chronic granulomatous inflammation and indurative pancreatitis can sometimes mimic a relapse on CT and PET/CT image tests.

## Introduction

A solid pseudopapillary tumor (SPT) is a rare pancreatic lesion that usually affects young and middle-aged patients and it has a female predominance and low malignant potential. Frantz VK first described it in 1959 [1]. The SPT is located throughout the pancreas but more frequently in the body or tail [2]. The clinical symptoms are usually non-specific. The most common are abdominal distention and pain. The lesion is usually well-demarcated and the standard treatment is surgical resection. The prognosis of SPT is usually very good after surgical excision; local recurrence has rarely been observed [3]. The major differential diagnosis of SPT is with neuroendocrine tumor/carcinoma and acinar cell carcinoma, cystadenomas, cystadenocarcinomas, intraductal papillary mucinous neoplasms, and teratomas. However, pancreatoblastoma should be excluded, too [4].

## Case presentation

**Figure 1:**
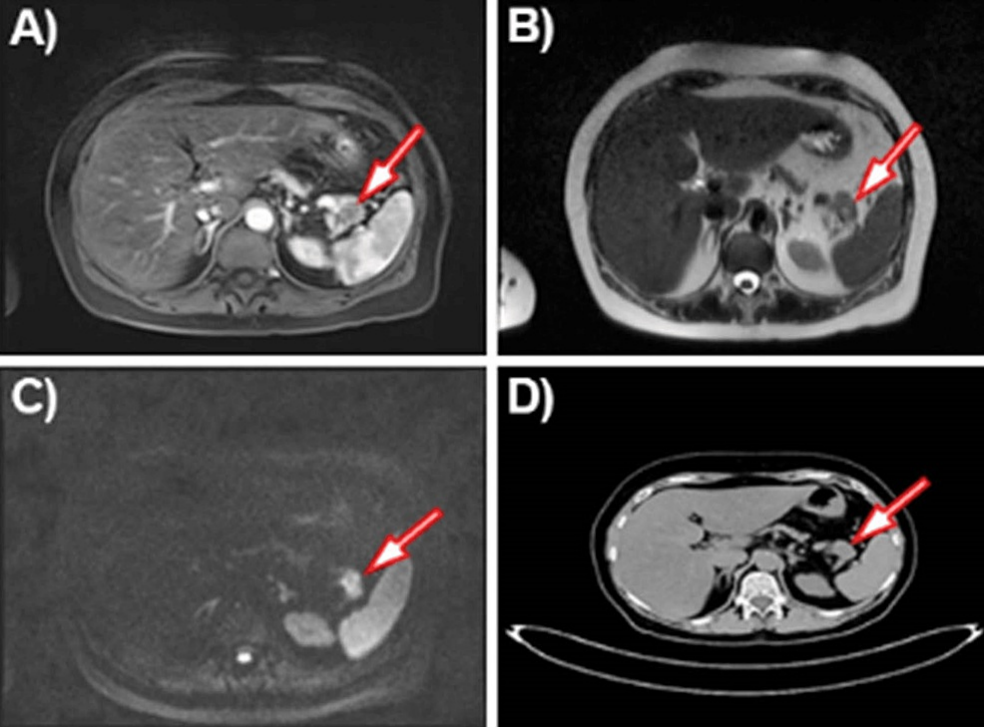
(A) MRI of the abdomen, T1 +contrast; (B) MRI of the abdomen T2; (C) MR DWI (diffusion); (D) Abdominal computed tomography showing tumor mass in the tail of the pancreas. MRI: magnetic resonance imaging, DWI: diffusion-weighted imaging

We present the case of a 60-year-old woman who complained of occasional abdominal pain for two months. The patient had no previous medical history of biliary pathology. She was admitted to the hospital for diagnosis and treatment. A magnetic resonance (MR) examination of the abdomen was performed with evidence of a tumor in the area of the pancreas's tail, near the spleen's hilum, measuring 22 mm by 21 mm by 19 mm, with outlines protruding beyond the contour of the organ. The lesion was slightly hyperintense at T2 and hypointense at T1, with restriction of diffusion, and it was relatively hypovascular on post-contrast images. The lesion was located near the lienal vein without convincing MR evidence of invasion. There was striation of the surrounding adipose tissue (Figure 1).

**Figure 2:**
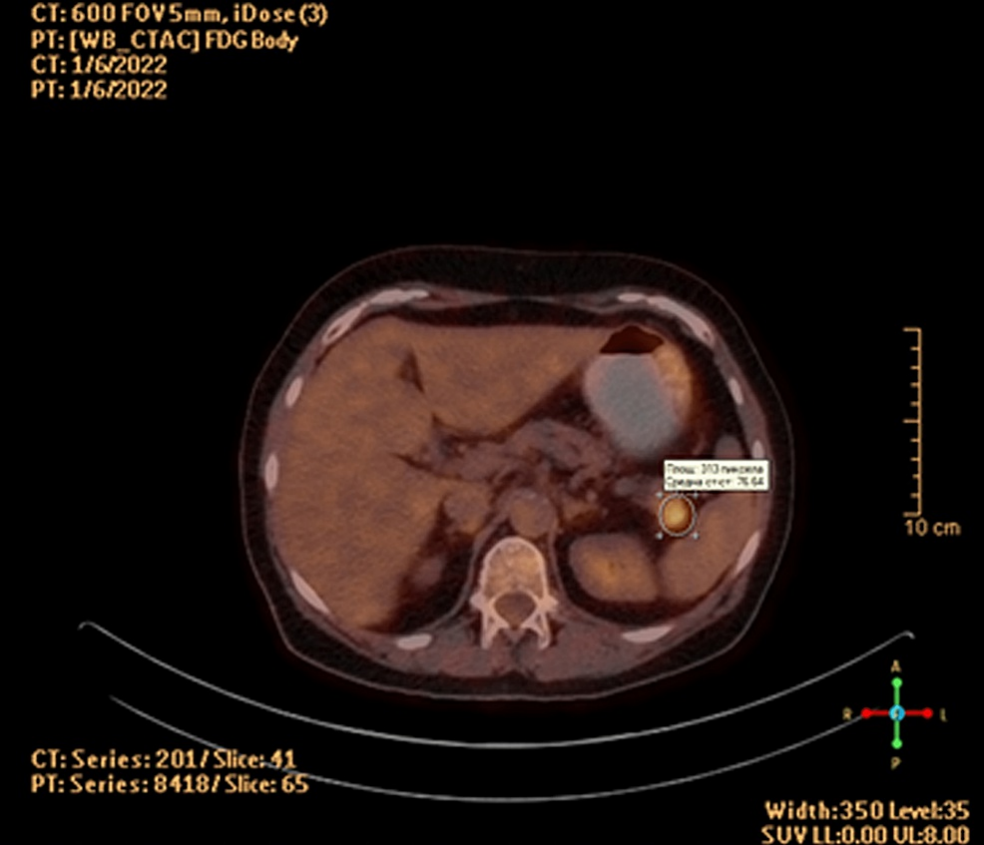
PET/CT of the patient PET/CT: positron emission tomography/computed tomography

Positron emission tomography/computed tomography (PET/CT) was also carried out and showed the presence of an isodense mass with pathological metabolism (12/14/19mm/SUVmax 6.5). There were no malignant lymphadenopathy or distant metastatic lesions found. The spleen was slightly enlarged (124 mm) (Figure 2).

The patient underwent computed tomography (CT)-guided percutaneous core needle biopsy to obtain cytologic and histologic analysis material. Unfortunately, the core biopsy was unsuccessful due to the patient's breathing-related movements. There was a small post-procedure iatrogenic hematoma around the tail of the pancreas and the spleen hilum.

The patient was discussed at the general clinical council, and based on her clinical and radiographic data, it was decided that she is indicated for surgical treatment to achieve excision of the tumor and histological investigation and diagnosis.

The patient underwent surgery four months after the onset of symptoms. The pancreatic tail was reached, and a large tumor formation with suspicious infiltration to the splenic hilum was found. A distal pancreatectomy with splenectomy was performed. Three drains were placed, which were removed on the third day. The postoperative period went uneventful.

**Figure 3:**
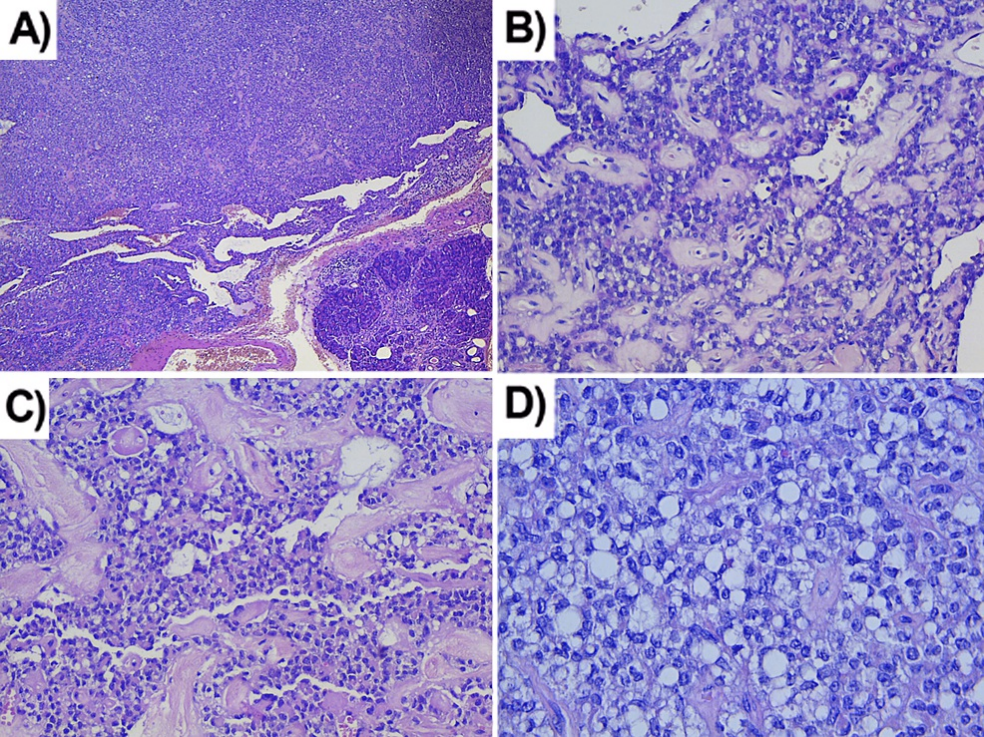
Histological characteristics of solid pseudopapillary neoplasm. (A) Solid areas (hematoxylin-eosin stain, magnification x40); (B) Zones with pseudopapillary pattern (hematoxylin-eosin stain, magnification x200); (C) Hyalinized stroma (hematoxylin-eosin stain, magnification x200); (D) Clear cell changes (hematoxylin-eosin stain, magnification x400).

The material was thoroughly examined after the whole tumor lesion was received in the pathology department. Material represented by the spleen measuring 14/7/3 cm, part of the pancreas measuring 6/7 cm, and surrounding adipose tissue measuring 15/6 cm. On a section in the pancreas, a tumor formation with a whitish color and dense consistency was found, located 6 cm from the surgical margin and 3 cm from the hilum of the spleen. The formation had approximate dimensions of 2.2/2.1/1.9 cm. Histologically, there was a relatively well-demarcated tumor formation comprises solid areas and zones with pseudopapillary growth of enlarged tumor cells (Figures 3A-3B).

Tumor cells were located around connective tissue areas with dilated small-caliber blood vessels. The cells have a moderately abundant cytoplasm and vacuoles found perinuclearly, with relatively uniform nuclei with indistinct nucleoli; in some of the nuclei, there were intranuclear grooves. Areas of cystic degeneration and others with fibrosis, hyalinosis, and foam cells were found in the stroma.

**Figure 4:**
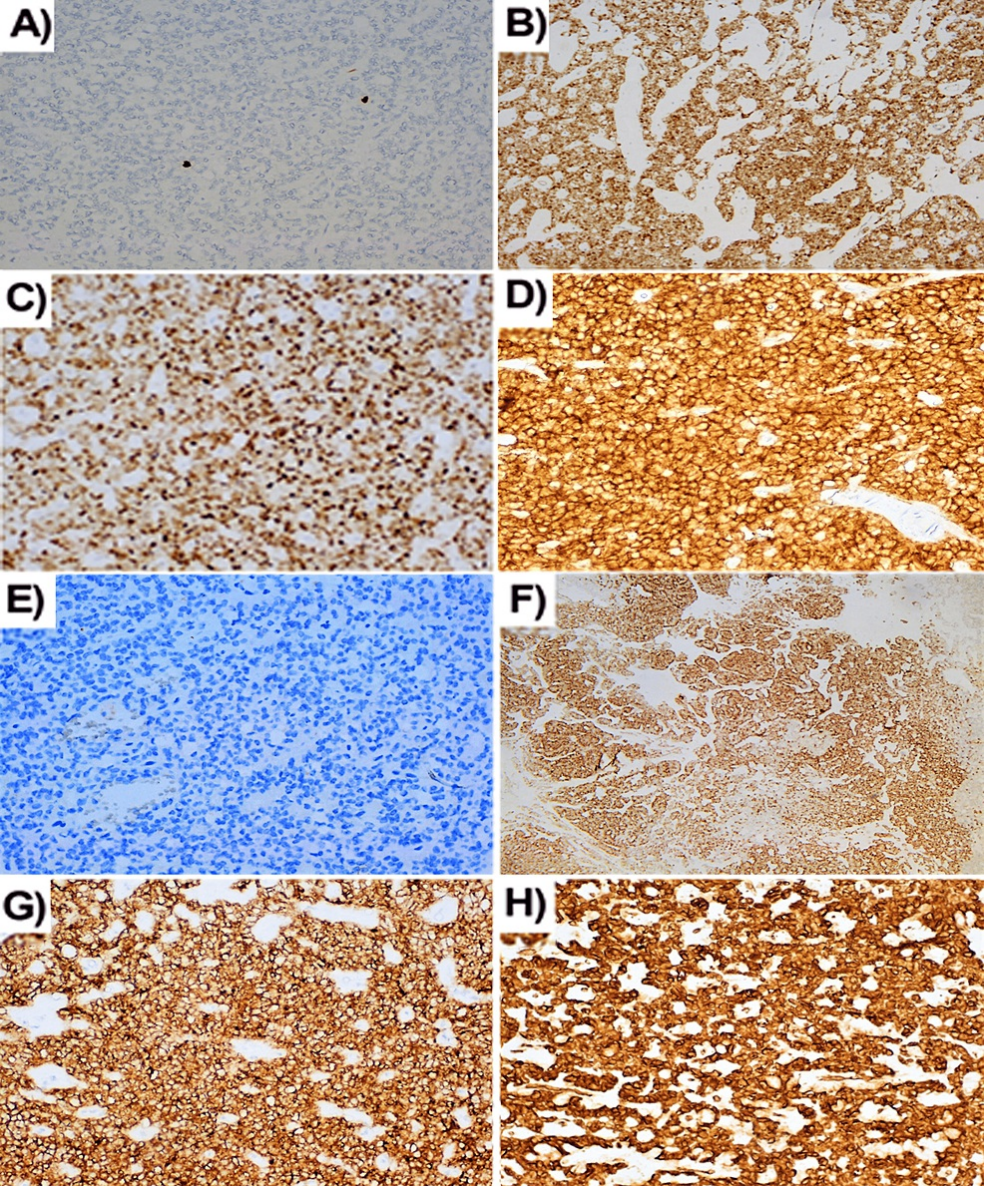
Immunohistochemical characteristics of solid pseudopapillary neoplasm. (A) Nuclear Ki-67 expression - low Ki-67 score (magnification x200); (B) Nuclear β-catenin expression (magnification x100); (C) Cyclin D1 expression (magnification x200); (D) CD56 expression (magnification x200); (E) Loss of e-cadherin expression (magnification x100); (F) Cytokeratin AE1/AE3 expression (magnification x40); (G) Membranous CD10 expression (magnification x100); (H) Vimentin expression (magnification x100).

Immunohistochemistry showed weak focal expression of chromogranin A in some of the cells. Focal expression of synaptophysin in tumor cells and marked expression in cells from the islets of Langerhans located in the periphery. Cytokeratin AE1/AE3 expression was observed focally in areas of papillary growth. Tumor cells diffusely and intensively express vimentin and CD56. Diffuse membranous expression was also observed with CD10 staining. CyclinD1 expression is moderately intense in tumor cells; beta-catenin has diffuse expression (Figure 4).

**Figure 5:**
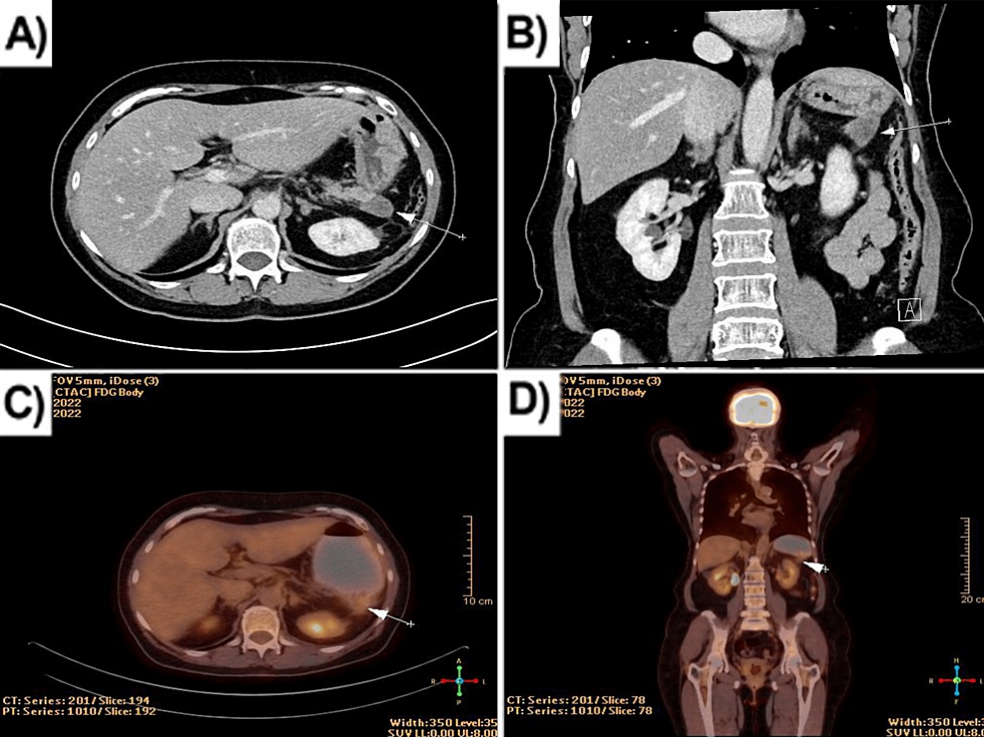
Image tests (five months after the operation). (A) Abdominal computed tomography (axial plane) showing a mass suspicious for recurrence; (B) Abdominal computed tomography (coronal plane); (C) PET/CT (axial plane); (D) PET/CT (coronal plane). PET/CT: positron emission tomography/computed tomography

The patient underwent four cycles of adjuvant chemotherapy with gemcitabine, which she tolerated well without complaints. In a control CT scan of a small pelvis (five months after the operation), a cystic lesion suspicious for local recurrence was visualized in the pancreatic tail - a rounded parenchymal lesion with a native density of 39HE and a post-contrast density of 45HE was found in the pancreatic tail, the fatty interface between the formation and the gastric wall was effaced, and a small amount of fluid was noted perifocally (Figure 5). Restaging the tumor with PET/CT showed evidence of a possible relapse - a 20/30mm cystic heterodense mass was visualized with a single area of increased radiopharmaceutical accumulation located peripherally with SUVmax 3.15. The finding was in contact with the gastric fundus, and infiltration could not be ruled out (Figures 5C-5D). That is why the patient underwent a second operation. A partial omentectomy was performed, and the omental bursa was opened - numerous adhesions from the previous surgery were found. A thorough debridement was performed. The greater curvature of the stomach was tightly attached to the body of the pancreas. A lesion with a dense consistency, measuring about 2 cm, was reached, located at the site of the resected tail of the pancreas. A part from the lesion was sent for rapid frozen section biopsy with a result for the granulomatous inflammatory process. A distal resection of the pancreas was performed until intact parenchyma was reached. One drain was placed in the bursa omentalis. Subsequent histological examination showed chronic indurative pancreatitis and areas with steatonecrosis, lipogranulomas, and fibrosis.

The patient is followed up every three months with abdominal CT and PET/CT. One year later, the patient had no complaints with good tolerance to the treatment.

## Discussion

The SPT is a rare pancreatic lesion that usually affects young and middle-aged patients and has a female predominance. Frantz VK first described this tumor in 1959 as a papillary tumor of the pancreas [1]. Several years later (1966), WHO reclassified it as a solid pseudopapillary neoplasm (SPN). In 2010, the tumor was reclassified again as an SPT of low-grade malignancy [5]. Still in the literature, the tumor is known by many names such as a solid-cystic tumor, solid-cystic acinar tumor, papillary-cystic tumor, solid papillary tumor, and SPN [6].

The histogenesis and exact origin of SPT is still unknown and remains elusive. There are different hypotheses in the literature. Some authors suggest that this lesion is derived from pancreatic duct cells [7,8]. However, the tumor cells do not show ductal character - the K-RAS mutation and CK-19, a marker for conventional adenocarcinoma, are absent [8]. Other researchers consider centroacinar origin [9]. Another hypothesis is related to the neural crest and the fact that the tumor may be derived from pluripotent stem cells of the genital ridges that become attached to the pancreas during embryogenesis [10]. Some scientists hypothesize that SPN could develop from primordial totipotent cells that would later differentiate into duct epithelial, acinar, or endocrine patterns [9]. There is inadequate evidence for each of these theories, and its precise histogenesis and origin are still unknown.

The SPT is located throughout the pancreas but more frequently in the body or tail in adults and more common in the head of the pancreas in children [2]. In our case, the lesion was located in the tail.

This tumor usually affects young women in the second and third decade of life, with an average age of 27.2 years, and less frequently in older women and males [5]. Given that the woman in our case was 60 years old, it is clear that older patients, not only young ladies, can potentially develop such tumors.

The clinical symptoms are usually nonspecific. The most common are abdominal distention and pain. Later, a non-tender upper abdominal mass can be palpated. Other symptoms, such as discomfort, nausea, vomiting, and early satiety, are related to an intra-abdominal mass effect. However, most cases are accidentally found through imaging [11].

Due to the vague symptoms and the accidental discovery, the tumor may manifest itself grossly as a bulky mass, ranging from 0.5 to 34.5 cm; with a mean diameter of 6 cm, SPN is usually well-demarcated, encapsulated, with variable amounts of solid and cystic patterns. Larger tumors have a variegated and friable cut surface and exhibit a fibrous pseudo capsule. Smaller lesions are typically more solid but less clearly defined. Larger specimens frequently exhibit cystic degeneration and hemorrhage. Occasionally, the tumor may reach surrounding structures like the duodenum [12].

SPN has a varied combination of solid and pseudopapillary regions histologically, making it a heterogeneous mass. Pseudopapillae is created when tumor cells detach from blood arteries and form fibrovascular stalks or rosette-like structures [12]. The tumor cells are monomorphic and typically have a moderate amount of eosinophilic cytoplasm with intracytoplasmic hyaline globules, which are PAS+ and diastase resistant; they are positive for alpha-1-antitrypsin. Another finding is the perinuclear vacuoles [13]. The nuclei are relatively uniform, oval, with finely granular chromatin and distinctive longitudinal grooves. Mitotic figures are rarely observed (0-6/20 HPF) with no atypical forms; the Ki-67 index is very low [6]. Hyalinization or signs of degeneration, such as hemorrhages, foam cells, calcification, and cholesterol clefts, are typically present in the stroma in varying degrees [14]. Despite SPN being anatomically well-demarcated, microscopic infiltration of the adjacent pancreatic tissue can be found. An unusual finding is the presence of psammoma bodies [6].

Immunohistochemically the tumor cells are positive for β-catenin, vimentin, α1-antitrypsin, α1-chymotrypsin, cyclin D1, CD10, and CD 56. There is a loss of membranous expression of E-cadherin. In some cases, there is immunoreactivity for neuron-specific enolase, synaptophysin, and cytokeratin [15]. Progesterone receptor presence is frequently found.

The standard treatment is surgical resection, which aims to be as conservative as possible. Nevertheless, enucleation or inadequate resection is linked to local recurrence, a poor prognosis, and death; the suggested margin is 3-5 mm [16]. En bloc, splenectomy is the procedure of choice most frequently in patients with tumor mass close to the splenic hilum and splenic arteries. Typically, lymphadenectomy is not advised [17].

Although the tumor can have locally aggressive characteristics, it has a low-grade malignant potential. Rarely, the tumor exhibits extremely aggressive behavior; the tumor's histological characteristics in these cases include diffuse growth pattern, extensive necrosis, prominent nuclear atypia, high mitotic count (35-70/50 high power fields), or sarcomatoid features [18]. Size greater than 5 cm, male gender, necrosis, cellular atypia, vascular invasion, perineural invasion, and invasion into adjacent structures are poor prognostic factors [19]. The Ki-67 index has been proposed as a marker for malignant potential; a low index value (5%) indicates slow tumor growth [20].

The prognosis of SPT is usually excellent after surgical excision, even when there is metastatic disease. In 15% of all cases, there are metastases, most of which are in the liver. Local recurrence has rarely been observed in the patients' long-term follow-up [3,4].

The major differential diagnosis of SPT is with neuroendocrine tumor/carcinoma and acinar cell carcinoma. Another tumor that should be excluded is pancreatoblastoma [4].

## Conclusions

SPT is a rare pancreatic tumor that most commonly affects young women. The exact pathogenesis is still unclear. Although the tumor has locally aggressive characteristics, the prognosis is excellent after surgical excision. Our case emphasizes that this tumor can occur not only in young women but also in older patients. Chronic granulomatous inflammation and indurative pancreatitis can sometimes mimic a relapse on CT and PET/CT image tests.

## References

[1] Frantz VK (1959). Atlas of tumor pathology. Section VII-Fascicles 27 and 28. Tumors of the Pancreas.

[2] Lee SE, Jang JY, Hwang DW, Park KW, Kim SW (2008). Clinical features and outcome of solid pseudopapillary neoplasm: differences between adults and children. Arch Surg.

[3] Eder F, Schulz HU, Röcken C, Lippert H (2005). Solid-pseudopapillary tumor of the pancreatic tail. World J Gastroenterol.

[4] Jiménez-Fuertes M, Ramírez-García JR, Ruiz-Tovar J, Díaz García G, Durán-Poveda M (2016). Solid pseudopapillary neoplasm of the pancreas. Cir Esp.

[5] Romero MD, Morales Cárdenas A, Cabrales Vázquez JE (2021). Frantz tumor in a 58 year old woman; case report and literature review. Ann Med Surg (Lond).

[6] Zalatnai A, Kis-Orha V (2020). Solid-pseudopapillary neoplasms of the pancreas is still an enigma: a clinicopathological review. Pathol Oncol Res.

[7] Lieber MR, Lack EE, Roberts JR Jr (1987). Solid and papillary epithelial neoplasm of the pancreas. An ultrastructural and immunocytochemical study of six cases. Am J Surg Pathol.

[8] Ueda N, Nagakawa T, Ohta T (1991). Clinicopathological studies on solid and cystic tumors of the pancreas. Gastroenterol Jpn.

[9] Matsunou H, Konishi F (1990). Papillary-cystic neoplasm of the pancreas. A clinicopathologic study concerning the tumor aging and malignancy of nine cases. Cancer.

[10] Terris B, Cavard C (2014). Diagnosis and molecular aspects of solid-pseudopapillary neoplasms of the pancreas. Semin Diagn Pathol.

[11] Dinarvand P, Lai J (2017). Solid pseudopapillary neoplasm of the pancreas: a rare entity with unique features. Arch Pathol Lab Med.

[12] La Rosa S, Bongiovanni M (2020). Pancreatic solid pseudopapillary neoplasm: key pathologic and genetic features. Arch Pathol Lab Med.

[13] Meriden Z, Shi C, Edil BH (2011). Hyaline globules in neuroendocrine and solid-pseudopapillary neoplasms of the pancreas: a clue to the diagnosis. Am J Surg Pathol.

[14] Navale P, Savari O, Tomashefski JF, Vyas M. (2023). Solid pseudopapillary neoplasm. https://www.pathologyoutlines.com/topic/pancreassolidpseudo.html.

[15] Uppin SG, Hui M, Thumma V (2015). Solid-pseudopapillary neoplasm of the pancreas: a clinicopathological and immunohistochemical study of 33 cases from a single institution in Southern India. Indian J Pathol Microbiol.

[16] Kodama R, Koh Y, Midorikawa H, Yokota Y, Saegusa H, Ushimaru H (2021). A case of recurrence of a solid pseudopapillary neoplasm of the pancreas effectively treated with proton beam radiotherapy. Clin J Gastroenterol.

[17] Rebai W, Ben Mahmoud A, Chammakhi A (2021). Management of solid and pseudopapillary tumors of the pancreas: about 3 case reports. J Gastrointest Cancer.

[18] Tang LH, Aydin H, Brennan MF, Klimstra DS (2005). Clinically aggressive solid pseudopapillary tumors of the pancreas: a report of two cases with components of undifferentiated carcinoma and a comparative clinicopathologic analysis of 34 conventional cases. Am J Surg Pathol.

[19] Del Chiaro M, Verbeke C, Salvia R (2013). European experts consensus statement on cystic tumours of the pancreas. Dig Liver Dis.

[20] Mat Zin AA, Shakir KA, Aminuddin AR (2012). Solid-pseudopapillary carcinoma: a case study and literature review. BMJ Case Rep.

